# Breast Neuroendocrine Carcinoma: Molecular Insights Beyond Histology

**DOI:** 10.1200/PO-26-00416

**Published:** 2026-08-13

**Authors:** Christopher J. Schwartz, Tanner Mack, William Travis, Hong Zhang, Nour Abuhadra, Risa Kiernan, Giacomo Montagna, Edi Brogi, Fresia Pareja, Hannah Y. Wen, Dara S. Ross

**Affiliations:** ^1^ Department of Pathology and Laboratory Medicine, Memorial Sloan Kettering Cancer Center, New York, NY; ^2^ Department of Medicine, Memorial Sloan Kettering Cancer Center, New York, NY; ^3^ Department of Surgery, Memorial Sloan Kettering Cancer Center, New York, NY

## Abstract

**PURPOSE:**

Primary breast neuroendocrine carcinomas (NECs) are rare, high-grade malignancies that are frequently grouped with invasive breast carcinomas with neuroendocrine differentiation (IBC-NED), despite uncertain biological equivalence. We sought to define the clinicopathologic, immunophenotypic, and genomic features of breast NEC and to determine whether they represent a biologically distinct entity.

**METHODS:**

We performed a retrospective analysis of 24 primary breast NECs and compared them with 28 grade-matched IBC-NEDs. Clinicopathologic features, treatment response, and outcomes were evaluated. Immunohistochemical profiling included neuroendocrine and breast lineage markers and retinoblastoma (Rb) expression. Targeted tumor-normal next-generation sequencing was performed in a subset of NEC (n = 10) and IBC-NED (n = 7) to characterize genomic alterations and pathway-level differences.

**RESULTS:**

Breast NECs were predominantly estrogen receptor (ER)–negative/human epidermal growth factor receptor-2–negative (63%) and frequently exhibited small cell morphology. Despite radiographic response to neoadjuvant chemotherapy, no patients achieved pathologic complete response. Immunophenotypically, NECs demonstrated diffuse neuroendocrine marker expression and frequent loss of Rb protein (93%), consistent with RB pathway disruption. Compared with IBC-NED, NECs showed significantly lower ER expression and reduced GATA3 positivity. Genomically, NECs were enriched for *TP53* alterations (90% *v* 33%, *P* < .05) and more frequently harbored *RB1* alterations (60% *v* 14%), with concurrent *TP53*/*RB1* alterations in 50% of cases. Alterations in the PI3K pathway were also observed in NECs. By contrast, IBC-NED demonstrated recurrent luminal-type copy-number alterations, including amplifications in *FGFR1* and *CCND1*.

**CONCLUSION:**

Breast NEC is characterized by a distinct clinicopathologic and molecular profile compared with grade-matched IBC-NED, defined by frequent *TP53* and *RB1* alterations, RB pathway disruption, and limited response to neoadjuvant therapy. These findings support classification of breast NEC as a biologically distinct subtype and highlight potential avenues for biomarker-driven therapeutic strategies in this rare malignancy.

## INTRODUCTION

Primary breast neuroendocrine carcinomas (NECs) are rare, high-grade malignancies characterized by distinctive neuroendocrine morphology and diffuse expression of neuroendocrine markers.^1,2^ In the 2019 WHO classification, breast neuroendocrine neoplasms were redefined to include well-differentiated neuroendocrine tumors (NETs), NECs, and invasive breast carcinomas (IBC) of no special type with neuroendocrine differentiation (IBC-NED), reflecting increasing recognition of biologic heterogeneity within this spectrum. Historically, however, many studies used broader terminology such as breast carcinoma with neuroendocrine differentiation, often grouping together morphologically distinct entities, including conventional IBC with only focal neuroendocrine marker expression. Definitions used in prior studies have therefore varied substantially across classification systems.

**CONTEXT**

**Key Objective**
To determine whether primary breast neuroendocrine carcinomas (NECs) are biologically distinct from invasive breast carcinomas with neuroendocrine differentiation (IBC-NED).
**Knowledge Generated**
Breast NECs show distinct clinicopathologic, immunophenotypic, and genomic features compared with IBC-NED, being predominantly estrogen receptor–negative/human epidermal growth factor receptor-2–negative, and demonstrating limited pathologic response to neoadjuvant chemotherapy despite radiographic tumor reduction. Genomically, NECs are enriched for *TP53* and *RB1* alterations, with frequent RB pathway disruption and recurrent PI3K pathway changes, whereas IBC-NED more commonly exhibits luminal-type copy-number alterations, including *FGFR1* and *CCND1 *amplifications.
**Relevance**
These findings support breast NEC as a biologically distinct subtype rather than a variant of IBC-NED. Integrated morphologic, immunophenotypic, and molecular evaluation may improve diagnostic accuracy and inform development of biomarker-driven therapeutic strategies for this rare and aggressive malignancy.

Although breast NECs comprise only a small subset of breast cancers, they are often associated with high-grade cytology, nodal involvement, and variable response to systemic therapy.^3-5^ Notably, there are currently no disease-specific treatment guidelines for breast NEC, and management is largely extrapolated either from conventional breast cancer regimens or from small cell neuroendocrine carcinoma protocols derived from extramammary sites, with uncertain efficacy. Distinguishing true NEC from high-grade IBC-NED remains diagnostically challenging because of overlapping morphologic and immunophenotypic features. However, published studies have reported molecular and clinicopathologic differences between NECs and high-grade IBC-NED that may have prognostic and therapeutic relevance.^6^

Histologically, breast NECs resemble extrapulmonary NECs, including small cell and large cell subtypes, and are commonly associated with alterations in the RB and p53 tumor suppressor pathways.^7-9^ Emerging genomic data suggest recurrent alterations in the PI3K signaling pathway, raising the possibility that breast NECs share molecular vulnerabilities with NECs at other anatomic sites. Despite increasing recognition of this entity, the integrated molecular landscape of breast NECs and the relationship between immunophenotypic features and underlying genomic alterations remain incompletely characterized, largely due to its rarity. In addition, potential neuroendocrine-associated therapeutic targets, including DLL3, have not been systematically evaluated in this context.^10^

To address these gaps, we conducted a retrospective, multimodal analysis of primary breast NECs, integrating clinicopathologic, immunohistochemical (IHC), and next-generation sequencing data. Using grade-matched IBC-NED as a comparator, we aimed to determine whether NEC constitutes a biologically distinct entity or falls along a spectrum of neuroendocrine-differentiated breast carcinomas. Specifically, we sought to define distinguishing molecular and immunophenotypic features, evaluate treatment response patterns, and identify candidate biomarkers to refine diagnostic classification and inform precision oncology strategies in this rare but aggressive subtype.

## METHODS

### Cases

After institutional review board approval (Protocol No. 17-287), we conducted a retrospective study of breast NEC identified from an institutional pathology database. All cases underwent centralized review by two breast pathologists (C.J.S. and D.S.R.) and one thoracic pathologist (W.T.) with expertise in pulmonary neuroendocrine neoplasms and cases were classified by consensus in accordance with World Health Organization criteria (5th Edition) and study definitions.^1^ Tumors were subclassified as small cell NEC (SCNEC), mixed SCNEC-ductal carcinoma, or large cell NEC (LCNEC). Classification was based on cytomorphologic features and IHC results. SCNEC was defined as a high-grade carcinoma composed of cells with high nuclear-to-cytoplasmic ratios, scant cytoplasm, finely granular chromatin, and inconspicuous nucleoli. LCNEC was characterized by large, pleomorphic cells with abundant cytoplasm, vesicular to coarse chromatin, and prominent nucleoli, consistent with features described in extra-mammary sites. Cases with admixed conventional invasive carcinoma were classified as mixed SCNEC-ductal carcinoma. IBC-NED was defined as invasive breast carcinoma demonstrating predominantly diffuse neuroendocrine morphology and neuroendocrine marker expression that did not fulfill established morphologic criteria for NEC. Conventional invasive carcinomas of no special type with only focal neuroendocrine marker expression and well-differentiated NET (grade 1 or 2) were excluded from the study cohort.

Estrogen receptor (ER), progesterone receptor (PR), and human epidermal growth factor receptor 2 (HER2) status were assessed using standardized clinical assays according to contemporaneous ASCO/CAP guidelines^11,12^ and centrally reviewed by study pathologists (C.J.S. and D.S.R.). In patients treated with neoadjuvant chemotherapy (NACT), tumor cellularity and residual cancer burden (RCB) index were centrally reassessed by a breast pathologist (C.J.S.) using established methods.^13^

### Immunohistochemistry

IHC analysis for retinoblastoma (Rb) and neuroendocrine markers (including synaptophysin, chromogranin, CD56, and INSM1) was conducted retrospectively and interpreted according to established protocols.^14^ NECs were further assessed for breast-lineage markers, including GATA3, SOX10, and TRPS1. Staining was performed using validated monoclonal antibodies: TRPS1 (EPR171671, Abcam), GATA3 (L50-823, Biocare Medical), and SOX10 (BC34, Biocare Medical), following standard methodologies.^15^ For TTF-1 and DLL3, IHC staining was performed on the Ventana Benchmark Ultra automated platform, using heat-induced antigen retrieval and OptiView detection (Ventana Medical Systems). DLL3 expression was considered positive if cytoplasmic and/or membranous staining was observed in >1% of tumor cells, based on previous protocols.^16^

### Next-Generation DNA Sequencing

A subset of cases underwent retrospective paired tumor-normal DNA targeted sequencing using the FDA-cleared MSK-IMPACT (Integrated Mutation Profiling of Actionable Cancer Targets) assay in a Clinical Laboratory Improvement Amendments–accredited laboratory.^17^ The assay involves hybridization capture and deep sequencing of all protein-encoding exons and selected introns of up to 505 key oncogenes and tumor suppressor genes, enabling detection of single-nucleotide variants, small insertions/deletions, copy-number alterations, and selected structural rearrangements. Overall, the targeted regions span approximately 1.2 megabases of the human genome. Genomic alterations in this study were additionally grouped into canonical oncogenic pathways, including TP53/RB, PI3K/AKT, receptor tyrosine kinase, and DNA repair pathways, for comparative analyses between the tumor groups.

### Statistics

Statistical analyses of clinical parameters and genomic alterations between groups were performed using R version 4.5.3. Fisher's exact test was used for categorical variables, including comparisons of mutation frequencies and copy-number alterations, while continuous variables were compared using the Mann-Whitney U test. All tests were two-sided, and statistical significance was defined as *P* < .05.

### Ethics Approval and Consent to Participate

The study was approved by the MSKCC institutional review board (Protocol No. 17-287) and performed in accordance with the Declaration of Helsinki. The requirement for written informed consent was waived as the study was retrospective, all data were deidentified, and the research contained no more than minimal risk to the participants or their privacy.

## RESULTS

### Clinicopathologic Features of Breast Neuroendocrine Carcinomas

Over a 20-year period, 24 primary breast NECs were identified, with findings summarized in Table 1. The median age at clinical presentation was 53.5 years (range, 33-95). The most common presenting symptoms included either palpable (50%, 12/24) or a mammographically detected mass (38%, 9/24). Metastatic disease at presentation was observed in 3 of 24 patients (12%), including two with a prior history of ER-positive invasive ductal carcinoma. Overall, 5 of 23 patients (22%) had a personal history of breast cancer; among these, one developed SCNEC at a previous lumpectomy site 24 months after an ER-positive invasive ductal carcinoma of no special type (IDC-NST; Appendix Table A1).

**TABLE 1.** Clinicopathologic Characteristics of Neuroendocrine Carcinoma Study Cohort

Parameter	Number (range or percent)
Median age at diagnosis, years (range)	53.5 (33-95)
Clinical presentation	
Palpable mass^a^	12/24 (50)
Mammographically detected mass	9/24 (38)
Metastatic disease	3/24 (12)
Personal breast cancer history	5/23 (22)
NEC histologic type	
Small cell	13/24 (54)
Large cell	7/24 (29)
Mixed small cell + ductal	4/24 (17)
Ductal carcinoma in situ identified	16/21 (76)
Biomarkers	
ER-/HER2-	15/24 (63)
ER+/HER2-	8/24 (33)
ER-/HER2+	1/24 (4)
Surgical treatment	21/24 (88)
Breast conservation	8/21 (38)
Mastectomy	13/21 (62)
Median untreated tumor size, mm (range)	30 (6-94)
Staging	
T1	7/21 (33)
T2	9/21 (43)
T3	3/21 (14)
Lymph node metastasis	10/19 (52)
NACT	9/21 (43)
Pathologic complete response	0/9 (0)

Abbreviations: ER, estrogen receptor; HER2, human epidermal growth factor receptor-2; NEC, neuroendocrine carcinoma; NACT, neoadjuvant chemotherapy.

^a^ One patient presented with a NEC at prior surgical site of an ER+ invasive ductal carcinoma.

**FIG 1.**
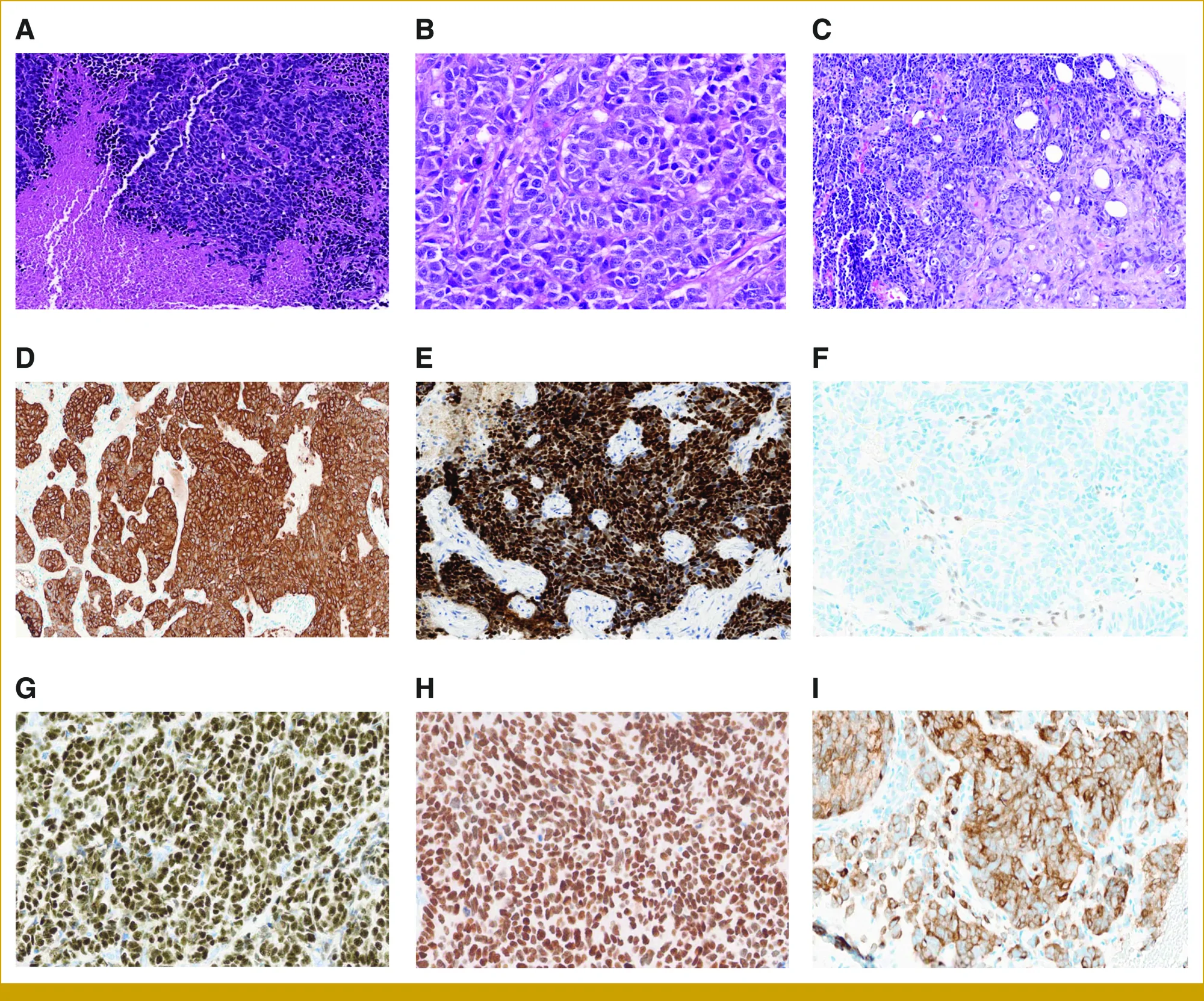
Histologic and IHC features of breast neuroendocrine carcinoma. (A) SCNEC showing sheets of tumor cells with high nuclear-to-cytoplasmic ratios and zonal necrosis (Case 1, H&E, 20×). (B) Large cell neuroendocrine carcinoma with a trabecular growth pattern, composed of pleomorphic tumor cells with abundant cytoplasm, vesicular chromatin, and prominent nucleoli (Case 18, H&E, 40×). (C) Mixed small cell neuroendocrine carcinoma and invasive ductal carcinoma (Case 24, H&E, 20×). (D) Diffuse, strong synaptophysin expression (Case 19, 20×) and (E) INSM1 expression in small cell neuroendocrine carcinoma (Case 1, 20×). (F) Loss of Rb expression in tumor cells (Case 19, 20×). (G) Nuclear GATA3 expression (Case 23, 40×) and (H) TRPS1 expression in tumor cells (Case 1, 20×). (I) DLL3 expression in tumor cells (Case 19, 40×). IHC, immunohistochemical; Rb, retinoblastoma; SCNEC, small cell neuroendocrine carcinoma.

Histologically, most NECs were classified as SCNEC (54%, 13/24), while 7 cases were large cell neuroendocrine carcinoma (LCNEC; 29%, 7/24) and 4 cases (17%, 4/24) showed a mixed pattern of SCNEC and IDC-NST (Table 1; Figs 1A, 1B, 1C). Ductal carcinoma in situ was identified in 16 of 21 cases (76%). Most tumors were ER-negative/HER2-negative (63%, 15/24), while one third were ER-positive, with staining ranging from 25% to 95%, and HER2-negative (33%, 8/24). One SCNEC (Case 20) was ER-negative/HER2-positive (4%, 1/24). Surgical management more frequently involved mastectomy (62%, 13/21) than lumpectomy (38%, 8/21). The median untreated tumor size was 30 mm (range, 6-94), with most tumors classified as T2 (43%, 9/21). Nodal involvement was identified in 52% (10/19) of patients at the time of surgery.

### Lack of Pathologic Complete Response to Neoadjuvant Therapy

NACT was administered in 43% (9/21) of patients, most commonly using platinum-based regimens, including combinations with etoposide or immunotherapy (Appendix Table A2). Despite radiographic tumor reduction in all evaluable cases (8/8), no pathologic complete responses were observed.

Residual disease was common, with 78% (7/9) of treated tumors classified as RCB class II (range, 1.4-4.3). Three patients remained node-positive at resection (all N1a). Notably, four cases showed no definitive histologic evidence of treatment response. These findings indicate limited pathologic response to neoadjuvant therapy across heterogeneous regimens.

Immunophenotypic Features Highlight RB Pathway Disruption

Most NECs (83%; 20/24) demonstrated strong and diffuse expression (>50% of tumor cells) of at least one neuroendocrine marker (Table 2 and Appendix Table A3). Synaptophysin was the most consistently expressed marker (92%, 22/24), followed by CD56 (90%, 9/10) and INSM1 (86%, 12/14), while chromogranin expression was less frequent (61%, 11/18; Figs 1D and 1E).

**TABLE 2.** IHC Characteristics of Primary Breast Neuroendocrine Carcinoma Cohort

Immunohistochemical Marker	Positive Cases, n/N (%)
Neuroendocrine marker expression	24/24 (100)
Nondiffuse/low (<50%)	4/24 (17)
Diffuse (>50%)	20/24 (83)
Neuroendocrine markers	
Synaptophysin	22/24 (92)
CD56	9/10 (90)
INSM1	12/14 (86)
Chromogranin	11/18 (61)
Additional markers	
Ki-67 proliferation index (>90%)	17/21 (81)
Rb loss	19/20 (93)
TRPS1	12/16 (75)
GATA3	13/24 (54)
DLL3	3/12 (25)
TTF-1	2/21 (10)
SOX10	1/18 (5)

Abbreviations: IHC, immunohistochemical; Rb, retinoblastoma.

Consistent with their high-grade biology, loss of Rb protein expression was observed in 93% of cases (19/20), and proliferative indices were markedly elevated, with Ki-67 exceeding 90% in most tumors (81%, 17/21).

Markers supporting breast lineage were retained in a substantial subset of cases, including TRPS1 (75%, 12/16) and GATA3 (54%, 13/24), while SOX10 expression was rare (5%, 1/18; Figs 1G and 1H). Aberrant expression of nonmammary markers was uncommon, including patchy, nondiffuse TTF-1 positivity in 10% (2/21). Notably, cytoplasmic and/or membranous expression of DLL3, a Notch pathway inhibitory ligand associated with pulmonary small cell carcinoma, was observed in 25% of evaluable cases (3/12; Fig 1I).

### Breast Neuroendocrine Carcinomas Differ From Grade-Matched IBC-NED

**FIG A1.**
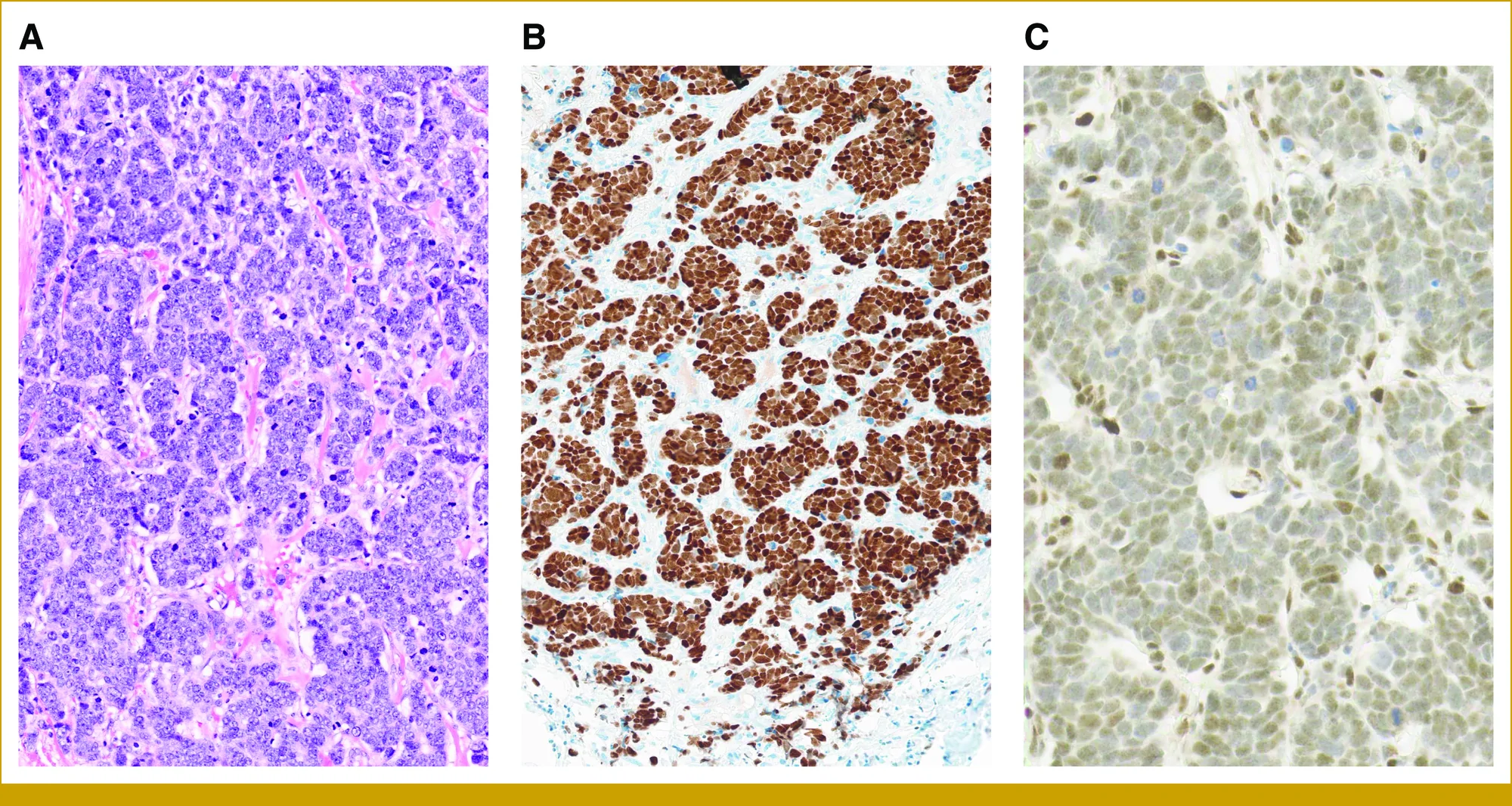
Histologic and IHC features of IBC-NED. (A) Representative histology (Case 20, H&E, 20×). (B) Diffuse and strong GATA3 staining (20×). (C) Retained Rb expression (40×). IBC-NED, invasive breast carcinoma with neuroendocrine differentiation; IHC, immunohistochemical.

Compared with grade-matched IBC-NED (n = 28; Appendix Fig A1; Appendix Table A4), NECs demonstrated distinct clinicopathologic and immunophenotypic features (Table 3). Median age at diagnosis was identical between groups (53.5 years), and tumor size was numerically larger in NECs (30 mm *v* 22 mm), although not statistically significant (*P* = .22).

**TABLE 3.** Comparative Analysis of Breast Neuroendocrine Carcinoma and IBC-NED

Parameter	NEC (n = 24)	IBC-NED (n = 28)	*P*
Median age at diagnosis, years (range)	53.5 (33-95)	53.5 (28-83)	1
Median untreated tumor size, mm (range)	30 (6-94)	22 (8-132)	.22
Biomarkers, n/N (%)			
ER+/HER2-	8/24 (33)	26/28 (92)	<.05
ER+/HER2+	1/24 (4)	2/28 (8)	1
ER-/HER2-	15/24 (63)	0/28 (0)	<.05
NE marker expression, n/N (%)			<.05
Nondiffuse/low (<50%)	4/24 (17)	0/28 (0)	
Diffuse (>50%)	20/24 (83)	28/28 (100)	
GATA3 expression, n/N (%)	13/24 (54)	15/15 (100)	<.05
Lymph node metastasis, n/N (%)	9/18 (50)	12/20 (60)	.75
NACT, n/N (%)	9/21 (43)	5/27 (19)	<.05
Pathologic complete response	0/9 (0)	0/5 (0)	1
Patients with clinical follow-up, n/N (%)	20/24 (83)	26/28 (93)	1
Median follow-up in months (range)	23 (6-168)	81 (49-192)	<.05
Adjuvant hormonal therapy, n/N (%)	8/18 (44)	26/26 (100)	<.05
Adjuvant chemotherapy, n/N (%)	15/18 (83)	22/26 (84)	1
Local recurrence, n/N (%)	3/20 (15)	1/26 (4)	1
Distant metastasis, n/N (%)	8/20 (40)	12/26 (46)	.76
Disease status, n/N (%)			
No evidence of disease	10/20 (50)	13/26 (50)	
AWD/DOD	10/20 (50)	9/26 (35)	

Abbreviations: AWD, alive with disease; DOD, death of disease; ER, estrogen receptor; HER2, human epidermal growth factor receptor-2; IBC-NED, invasive breast carcinoma with neuroendocrine differentiation; NACT, neoadjuvant chemotherapy; NEC, neuroendocrine carcinoma; NE, neuroendocrine.

NECs were significantly less likely to be ER-positive/HER2-negative (33% vs 92%, *P* < .05) and were predominantly ER-negative/HER2-negative (63% *v* 0%, *P* < .05). Although diffuse neuroendocrine marker expression was observed in both groups, focal or low-level expression (<50%) occurred only in NECs (17% *v* 0%, *P* < .05). GATA3 expression was also significantly less frequent in NECs (54% *v* 100%, *P* < .05).

Rates of nodal involvement were similar (50% *v* 60%). Patients with NEC were more likely to receive NACT (43% *v* 19%, *P* < .05), reflecting receptor-negative disease. Despite differing treatment approaches, no pathologic complete responses were observed in either cohort.

In the adjuvant setting, patients with IBC-NED were more likely to receive endocrine therapy (100% *v* 44%, *P* < .05), while chemotherapy use was similar. Clinical outcomes appeared broadly comparable, although interpretation is limited by shorter follow-up in the NEC cohort (median, 23 *v* 82 months). At last follow-up, 50% of patients in each group were disease-free; however, disease-specific mortality was higher in NEC (50% *v* 35%).

### Recurrent *TP53 *and *RB1* Alterations Define the Genomic Landscape

**FIG 2.**
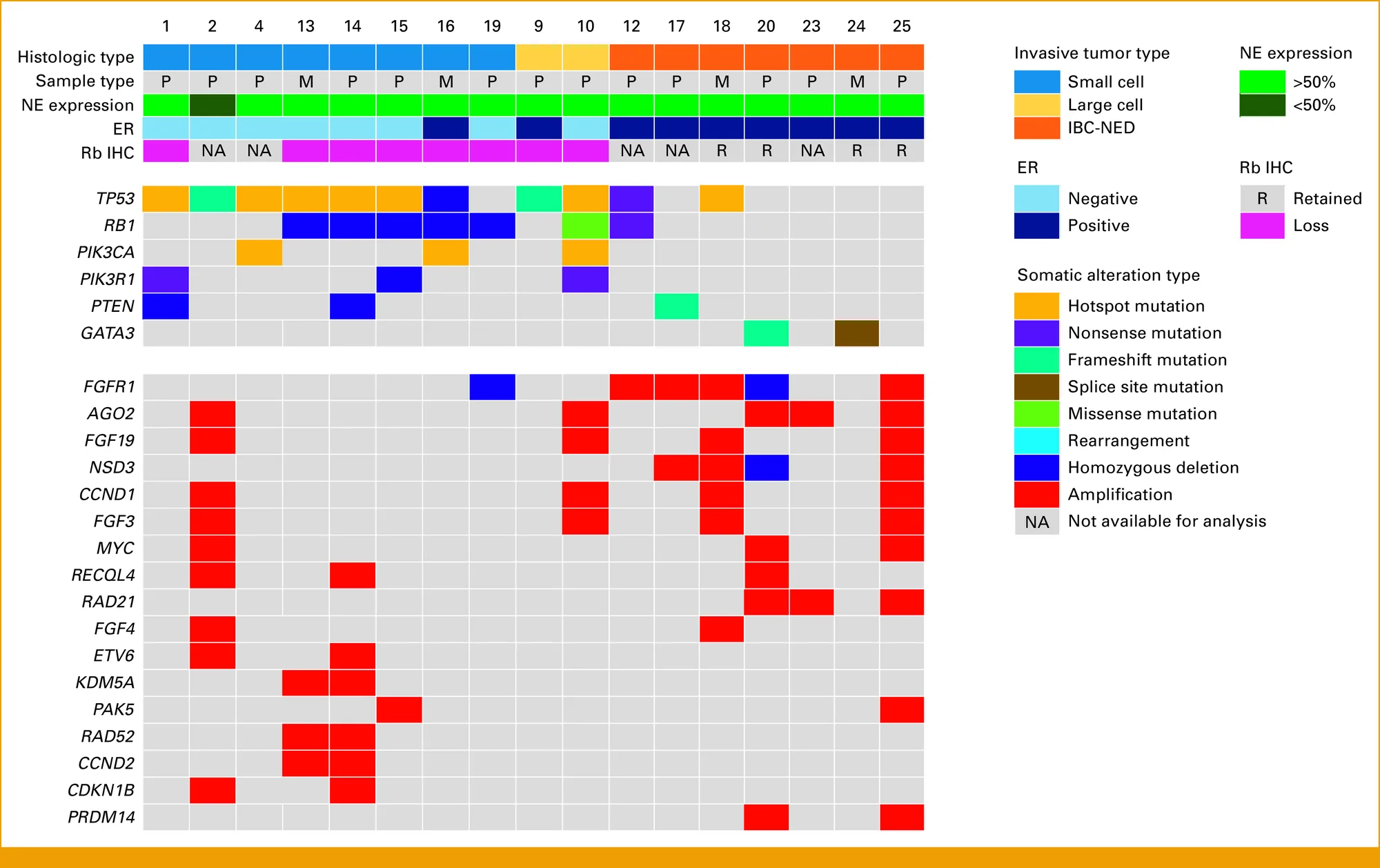
Genomic alterations in breast neuroendocrine carcinoma and IBC-NED. Oncoplot depicting genomic alterations identified in breast NEC and IBC-NED. Individual cases are represented in columns and genes in rows. Phenotype bars (top) indicate tumor type, expression of neuroendocrine IHC markers, estrogen receptor and Rb protein status (by immunohistochemistry). Somatic alterations are color-coded according to the legend (bottom right). ER, estrogen receptor; IBC-NED, invasive breast carcinoma with neuroendocrine differentiation; IHC, immunohistochemical; M, metastatic tumor; NE, neuroendocrine; P, primary tumor; Rb, retinoblastoma.

Paired tumor-normal DNA targeted results were retrospectively analyzed in a subset of NEC (n = 10; eight primary and two metastatic tumors), including SCNEC (n = 8) and LCNEC (n = 2). The most frequent genomic alterations involved *TP53* (90%, 9/10) and *RB1* (60%, 6/10), with concurrent *TP53*/*RB1* coalterations observed in 50% of cases (5/10; Fig 2).

Alterations affecting the PI3K pathway were also recurrent, including hotspot mutations in *PIK3CA* (30%, 3/10) and *PIK3R1* (30%, 3/10), as well as homozygous deletion of *PTEN* (20%, 2/10). These alterations were largely mutually exclusive. Additional recurrent events were infrequent.

### Distinct Genomic Profiles Between NEC and IBC-NED

Comparative genomic analysis with IBC-NED (n = 7) demonstrated distinct molecular profiles. NECs exhibited a significantly higher frequency of *TP53* alterations (90% *v* 33%, *P* < .05) and exclusive enrichment of PI3K pathway alterations (60% *v* 0%, *P* < .05).

*RB1* alterations were more frequent in NEC (60% *v* 14%), although this difference did not reach statistical significance (*P* = .13). Importantly, *RB1* alterations were associated with complete loss of Rb protein expression by immunohistochemistry (100%, 8/8 *v* 0/4; *P* < .05), consistent with functional inactivation of the RB pathway (Fig 2 and Table 4).

**TABLE 4.** Comparative Genomic Analysis of Breast Neuroendocrine Carcinoma and IBC-NED

Category	Alteration	NEC (n = 10), n (%)	IBC-NED (n = 7), n (%)	*P*
TP53/Rb	*TP53* alterations	9 (90)	2 (33)	<.05
	*RB1* alterations	6 (60)	1 (14)	.13
	*TP53*/*RB1* coalteration	5 (50)	1 (14)	.30
	Rb protein loss (IHC)^a^	8 (100)	0 (0)	<.05
PI3K pathway	*PIK3CA* mutation	3 (30)	0 (0)	.23
	*PIK3R1* mutation	3 (30)	0 (0)	.23
	PI3K pathway alteration (overall)	6 (60)	0 (0)	<.05
Copy-number alterations	*FGFR1* amplification	0 (0)	4 (56)	<.05
	*AGO2* amplification	1 (10)	4 (56)	.10
	*NSD3* amplification	0 (0)	3 (43)	.05
	*FGF19* amplification	1 (10)	3 (43)	.25
	*CCND1* amplification	1 (10)	3 (43)	.25
	*FGF3* amplification	1 (10)	3 (44)	.25
Genomic architecture	Recurrent amplicons (8p11, 11q13)	1 (10)	4 (56)	.10

Abbreviations: IBC-NED, invasive breast carcinoma with neuroendocrine differentiation; IHC, immunohistochemical.

^a^ For NEC, n = 8. For IBC-NED, n = 4.

By contrast, IBC-NED had fewer mutations and, in some cases, exhibited recurrent copy-number gains commonly seen in luminal breast cancers, such as amplifications involving *FGFR1*, *NSD3*, *CCND1*, and related loci (Fig 2 and Table 4). These copy-number changes were generally absent or rare in NECs (0%-10%).

Collectively, these findings demonstrate that NECs are characterized by a genomic profile defined by *TP53* and *RB1* alterations with recurrent PI3K pathway involvement, whereas IBC-NED more closely resembles high-grade luminal breast carcinoma.

## DISCUSSION

Primary breast NECs are rare, high-grade malignancies that remain incompletely defined at both the clinicopathologic and molecular levels.^1^ In this study, we demonstrate that breast NECs exhibit a constellation of features that distinguish them from grade-matched IBC-NED, supporting their classification as a biologically distinct entity rather than a high-grade variant within a spectrum of NED.^6^

A central finding of this study is the high prevalence of RB pathway disruption in NEC, evidenced by near-uniform loss of Rb protein expression and frequent *RB1* alterations. This was accompanied by a high rate of *TP53* mutations, with concurrent *TP53*/*RB1* alterations observed in a substantial subset of cases. Together, these findings parallel the genomic architecture described in NECs arising at other anatomic sites^7,8^ and support a model in which breast NEC is defined by dysregulation of key tumor suppressor pathways.^9^ By contrast, IBC-NED more commonly retained features of luminal breast carcinomas, including ER expression and recurrent copy-number gains such as *FGFR1* and *CCND1*, highlighting divergent oncogenic programs.

The immunophenotypic profile further reinforces this distinction. NECs demonstrated frequent and often diffuse expression of neuroendocrine markers, consistent with prior reports^2,11^ while exhibiting reduced expression of lineage-associated markers such as GATA3. Notably, loss of Rb expression was highly prevalent in NEC and correlated with underlying *RB1* alterations, suggesting functional inactivation of the RB pathway. Although this feature may have diagnostic utility, its role in routine practice remains to be established. Importantly, a subset of NECs demonstrated heterogeneous or only focal neuroendocrine marker expression despite otherwise characteristic morphologic features. This finding highlights a potential diagnostic pitfall, as reliance on the extent of neuroendocrine marker staining alone may contribute to misclassification of NEC. Accordingly, diagnosis should incorporate both morphologic and immunophenotypic findings in the appropriate clinicopathologic context. Occasional expression of nonmammary markers, including TTF-1, underscores a recognized diagnostic pitfall and highlights the importance of clinicopathologic correlation in excluding metastatic disease.^18,19^ Indeed, metastases to the breast from pulmonary and other extrapulmonary NECs represent an important consideration in this differential diagnosis.^20^

From a clinical perspective, NECs in this cohort were predominantly ER-negative/HER2-negative and were more frequently treated with NACT, often incorporating platinum-based regimens. Despite consistent radiographic responses, no pathologic complete responses were observed, and most tumors demonstrated moderate residual disease. Although interpretation is limited by treatment heterogeneity and sample size, these findings raise the possibility that breast NEC may exhibit relative resistance to currently used systemic therapies, including both conventional breast cancer regimens and neuroendocrine-directed approaches.^21,22^ Although responses to etoposide-based regimens have been reported,^23^ the optimal treatment strategy for breast NEC remains undefined.

The genomic landscape identified in NEC suggests potential avenues for therapeutic investigation. Recurrent alterations in *TP53* and *RB1*, while not directly targetable, define a molecular context that may influence response to specific therapeutic classes. In addition, recurrent PI3K pathway alterations observed in this cohort may represent a potential targetable vulnerability, although their clinical relevance in breast NEC remains to be determined. The identification of DLL3 expression in a subset of tumors is also of interest, given the development of DLL3-targeted therapies in neuroendocrine malignancies^10,24^; however, the variability in expression and limited sample size warrant cautious interpretation.

Several observations in this cohort raise broader biological questions. The occurrence of NEC in patients with a prior history of breast carcinoma, including cases arising at prior tumor sites, suggests the possibility of lineage plasticity or transdifferentiation, a phenomenon increasingly recognized in high-grade NECs, particularly in the setting of therapeutic pressure.^14^ Although these observations are intriguing, the current study was not designed to assess clonal relationships, and further investigation will be required to clarify these mechanisms.

This study has several limitations. The retrospective design and relatively small cohort size, particularly for genomic analyses, limit statistical power and generalizability. Treatment regimens were heterogeneous and not standardized, complicating assessment of therapeutic response. In addition, differences in follow-up duration between NEC and IBC-NED cohorts constrain comparative outcome analyses. Finally, classification of neuroendocrine neoplasms of the breast remains an evolving area, and although cases were centrally reviewed using current criteria,^1^ some degree of diagnostic subjectivity is unavoidable.

In summary, breast NEC is characterized by a combination of clinicopathologic, immunophenotypic, and molecular features that distinguish it from grade-matched IBC-NED. Frequent *TP53* and *RB1* alterations, RB pathway disruption, and recurrent PI3K pathway involvement define its molecular profile and align it more closely with NECs arising at other sites than with conventional breast carcinomas. These findings support classification of breast NEC as a biologically distinct subtype and highlight the need for prospective, multi-institutional studies to validate these observations and to develop biomarker-driven therapeutic strategies for this rare and aggressive malignancy.

## Data Availability

A data sharing statement provided by the authors is available with this article at DOI https://doi.org/10.1200/PO-26-00416. The data generated in this study are available from the corresponding author upon reasonable request.

## APPENDIX

**TABLE A1.** Clinicopathologic Features of Primary Breast Neuroendocrine Carcinoma Cohort

Case	Age (years)	Clinical Presentation	Germline Testing	Personal Cancer History (age, years)	Histology	Surgery	Nottingham Grade	Tumor Size (mm)	NACT	T Stage	Lymph Nodes	N Stage	ER	HER2	Adjuvant HT	Adjuvant CT	Adjuvant RT	Follow-Up (months)	Disease Status	Local Recurrence	Distant Metastasis (site)
1	78	Palpable mass	X	Bladder (remote); breast (synchronous DCIS, 78)	NEC (small cell)	TM	3	38	Yes	T2	Positive	N1a	Negative	Negative	No	Yes	No	30	DOD	No	Yes (brain)
2	45	Screening	X	None	NEC (small cell)	TM	3	26	Yes	T2	Negative	N0	Negative	Negative	No	No	Yes	31	No disease	Yes (axilla, 6 months)	No
3	51	Screening	X	None	NEC (large cell)	TM	3	6	No	T1b	Negative	N0	Positive	Negative	Yes	Yes	No	18	No disease	No	No
4	63	Palpable mass	X	None	NEC (small cell)	BCS	3	14	Yes	T1c	Negative	N0	Negative	Negative	Yes	Yes	No	21	DOD	No	Yes (brain)
5	79	Presented with metastasis	X	Breast (75)	NEC (small cell)	TM (for prior breast ca)	3	X	No	X	X	NX	Negative	Negative	No	No	No	168	DOD	No	Yes (lung)
6	59	Screening	Negative	None	NEC (large cell)	BCS	3	24	Yes	T2	Negative	N0	Negative	Negative	No	Yes	Yes	67	No disease	No	No
7	39	Palpable mass	Negative	None	NEC (large cell)	TM	3	57	No	T3	Positive	N1a	Positive	Negative	Yes	Yes	Yes	20	No disease	No	No
8	59	Palpable mass	Negative	None	NEC (small cell)	TM	3	9	Yes	T1c	Negative	N0	Negative	Negative	No	Yes	No	22	No disease	No	None
9	33	Palpable mass	Negative	None	NEC (large cell)	TM	3	94	No	T3	Positive	N3a	Positive	Negative	Yes	Yes	No	52	DOD	No	Yes (liver)
10	54	Screening	Negative	None	NEC (large cell)	TM	3	27	No	T2	Negative	N0	Negative	Negative	No	Yes	No	65	No disease	No	No
11	64	Screening	X	None	NEC (large cell)	BCS	3	14	No	T1c	Negative	N0	Negative	Negative	No	Yes	Yes	120	No disease	No	No
12	95	Palpable mass	X	None	NEC (small cell)	TM	3	76	No	T3	Positive	N1a	Negative	Negative	No	No	No	6	DOD	No	Yes (axillary lymph node)
13	53	Screening	X	None	NEC (small cell)	BCS	3	31	No	T2	Positive	N1mi	Negative	Negative	No	Yes	No	20	DOD	No	Yes (lung)
14	43	Screening	Negative	None	NEC (small cell)	BCS	3	30	No	T2	Positive	N1a	Negative	Negative	No	Yes	Yes	24	No disease	No	No
15	45	Screening	Negative	None	NEC (small cell)	TM	3	35	No	T2	Positive	N2a	Negative	Negative	No	Yes	No	20	AWD	Yes (chest wall, 20 months)	X
16	53	Presented with metastasis	X	Breast (41)	NEC (small cell)	TM (for prior breast ca)	3	X	No	X	X	X	Positive	Negative	Yes	No	Yes	156	AWD	No	Yes (brain, lung)
17	63	Palpable mass	Negative	Unknown	Mixed IDC + NEC (small cell)	BCS	3	30	No	T2	Positive	N3a	Positive	Negative	Yes	Yes	Yes	24	AWD	No	Yes (liver)
18	57	Palpable mass	Negative	None	NEC (large cell)	TM	3	35	Yes	T2	Negative	N0	Negative	Negative	X	X	X	X	X	X	X
19	44	Palpable mass	Negative	None	NEC (small cell)	None	3	X	X	X	X	X	Negative	Negative	X	X	X	X	X	X	X
20	57	Palpable mass	Negative	None	NEC (small cell)	BCS	3	8	Yes	T1a	Positive	N1a	Negative	Positive	Yes	Yes	Yes	22	No disease	No	No
21	51	Palpable mass	X	Breast (49)	NEC (small cell)	TM	3	17	No	T1c	X	Nx	Positive	Negative	Yes	Yes	Yes	15	No disease	Yes (breast, 24 months)	No
22	47	Screening	Negative	None	Mixed IDC + NEC (small cell)	BCS	3	12	Yes	T1c	Negative	N0	Positive	Negative	X	X	X	X	X	X	X
23	71	Palpable mass	*BRCA2*	None	Mixed IDC + NEC (small cell)	TM	3	44	Yes	T2	Positive	N1a	Positive	Negative	X	X	X	X	X	X	X
24	50	Presented with metastasis	Negative	Breast (43)	Mixed IDC + NEC (small cell)	None	3	X	X	X	X	X	Negative	Negative	X	X	X	6	DOD	No	Yes (bone, liver)

Abbreviations: AWD, alive with disease; BCS, breast-conserving surgery; CT, chemotherapy; DOD, died of disease; ER, estrogen receptor; HER2, human epidermal growth factor receptor-2; HT, hormonal therapy; IDC, invasive ductal carcinoma; NACT, neoadjuvant chemotherapy; NEC, neuroendocrine carcinoma; RT, radiotherapy; TM, total mastectomy; X, test not available/performed.

**TABLE A2.** NACT Response in NECs

Case	Histotype	NACT Regimen	Before Imaging Size (mm)	After Imaging Size (mm)	Pathologic Response	Tumor Size (mm)^a^	LN Status	Post-Treatment Cellularity (%)	RCB Burden	RCB Class
1	SCNEC	C + E	NA	NA	No	38	Positive	80	4.3	III
2	SCNEC	AC + IT	48	33	Yes	26	Negative	20	1.8	II
4	SCNEC	C + E	34	21	Yes	14	Negative	10	1.4	II
6	LCNEC	AC-T	NA	NA	No	24	Negative	80	2.3	II
8	SCNEC	C + E	18	14	Yes	9	Negative	20	1.5	II
18	LCNEC	CIS + E + IT	39	24	No	35	Negative	70	2.4	II
20	SCNEC	AC-THP	34	16	Yes	8	Positive	10	2.6	II
22	Mixed SCNEC + IDC	CIS + E	22	18	No^b^	12	Negative	80	2.1	II
23	Mixed SCNEC + IDC	C + E	26	16	Yes	44	Positive	30	3.7	III

Abbreviations: AC, doxorubicin and cyclophosphamide; C, carboplatin; CIS, cisplatin; E, etoposide; IDC, invasive ductal carcinoma; IT, immunotherapy; LCNEC, large cell neuroendocrine carcinoma; LN, lymph node; NA, not available; NACT, neoadjuvant chemotherapy; NECs, neuroendocrine carcinomas; RCB, residual cancer burden; SCNEC, small cell neuroendocrine carcinoma; THP, Herceptin/trastuzumab + pertuzumab.

a Tumor size at pathologic examination.

^b^ Only ductal component showed pathologic response.

**TABLE A3.** IHC Characteristics of Primary Breast Neuroendocrine Carcinoma Cohort

Case	Histology	ER (%)	HER2	Ki-67	NE Markers	Synaptophysin	Chromogranin	CD56	INSM1	GATA3	TTF-1	TRPS1	SOX10	Rb IHC	DLL4
1	NEC (small cell)	Negative	Negative	X	Positive	Positive	Negative	Negative	Negative	Negative	Positive (focal)	Positive	Negative	Loss	X
2	NEC (small cell)	Negative	Negative	>90%	Low positive	Positive	Positive	Positive	X	Negative	Negative	Negative	Negative	X	Negative
3	NEC (large cell)	Positive (60%)	Negative	>90%	Positive	Positive	Positive	X	X	Positive	X	Positive	X	Loss	Negative
4	NEC (small cell)	Negative	Negative	X	Positive	Positive	X	Positive	X	Positive (focal)	X	X	X	X	X
5	NEC (small cell)	Negative	Negative	X	Positive	Positive	Negative	X	X	Positive	Negative	X	X	X	X
6	NEC (large cell)	Negative	Negative	>90%	Positive	Positive	Negative	Positive	Positive	Positive (focal)	Negative	X	Negative	Retained	X
7	NEC (large cell)	Positive (90%)	Negative	>90%	Positive	Positive	Positive	X	Positive	Positive	Negative	Positive (focal)	Negative	Loss	Positive
8	NEC (small cell)	Negative	Negative	>90%	Positive	Positive	X	Positive	Positive	Negative	Negative	Positive	X	Loss	X
9	NEC (large cell)	Positive (95%)	Negative	>90%	Positive	Positive	Positive	X	X	Positive	Negative	X	X	Loss	X
10	NEC (large cell)	Negative	Negative	>70%	Positive	Positive	Positive	X	Positive	Negative	Negative	X	Negative	Loss	X
11	NEC (large cell)	Negative	Negative	>90%	Positive	Negative	Negative	Positive	X	Negative	Negative	Positive	Negative	Loss	Negative
12	NEC (small cell)	Negative	Negative	>90%	Low positive	Positive	Positive	X	X	Negative	Negative	Negative	Negative	Loss	Negative
13	NEC (small cell)	Negative	Negative	>95%	Positive	Positive	Positive	X	Positive	Positive	Negative	X	Negative	Loss	X
14	NEC (small cell)	Negative	Negative	>95%	Positive	Positive	Positive	Positive	Positive	Negative	Negative	Positive	Negative	Loss	X
15	NEC (small cell)	Negative	Negative	>90%	Positive	Positive	X	X	Positive	Positive (focal)	Negative	Negative	Negative	Loss	X
16	NEC (small cell)	Positive (80%)	Negative	70%	Positive	Positive	Negative	X	X	Positive	Negative	Positive	Negative	Loss	X
17	Mixed IDC + NEC (small cell)	Positive (50%)	Negative	60%	Positive	Positive	X	X	X	Positive	X	X	X	Loss	X
18	NEC (large cell)	Negative	Negative	>90%	Low positive	Positive	X	X	Positive	Positive (focal)	Negative	Positive	Negative	Loss	X
19	NEC (small cell)	Negative	Negative	>90%	Positive	Positive	Positive	Positive	Positive	Negative	Positive (focal)	Negative	Negative	Loss	Positive
20	NEC (small cell)	Negative	Positive	90%	Positive	Positive	Positive	Positive	X	Negative	Negative	Positive	Negative	Loss	Negative
21	NEC (small cell)	Positive (20%)	Negative	90%	Low positive	Negative	Negative	X	Positive	Negative	Negative	Positive	Positive	Loss	Negative
22	Mixed IDC + NEC (small cell)	Positive (25%)	Negative	>90%	Positive	Positive	Positive	Positive	Positive	Positive	Negative	Positive (ductal component only)	Negative	Loss	Negative
23	Mixed IDC + NEC (small cell)	Positive (95%)	Negative	50%	Positive	Positive	X	X	Negative	Positive	Negative	Positive	Negative	Loss	Negative
24	Mixed IDC + NEC (small cell)	Negative	Negative	>90%	Positive	Positive	Negative	X	Positive	Negative	Negative	X	Negative	X	Positive

Abbreviations: ER, estrogen receptor; HER2, human epidermal growth factor receptor; IHC, immunohistochemical; NE, neuroendocrine; NEC, neuroendocrine carcinoma; X, not available/not performed.

**TABLE A4.** Clinicopathologic Features of IBC-NED

Case	Age (years)	Clinical Presentation	Germline Testing	Histology	Surgery	Nottingham Grade	Tumor Size (mm)	NACT	T Stage	Lymph Nodes	N Stage	ER	HER2	NE Markers	GATA3	Adjuvant HT	Adjuvant CT	Adjuvant RT	Follow-Up (months)	Disease Status	Local Recurrence	Distant Metastasis (site)
1	45	Palpable mass	X	IBC with NE features	TM	3	19	Yes	T1c	Positive	N3a	Positive	Negative	Positive (SYN, CHR)	X	Yes	No	Yes	192	No disease	No	No
2	62	Screen detected	X	IBC with NE features	BCS	3	13	No	T1c	Positive	N1a	Positive	Negative	Positive (SYN, CHR)	X	Yes	Yes	Yes	56	No disease	No	No
3	53	Palpable mass	X	IBC with NE features	BCS	3	25	No	T2	Positive	N1a	Positive	Negative	Positive (SYN, CHR)	X	Yes	Yes	Yes	91	No disease	No	No
4	28	Palpable mass	Negative	IBC with NE features	TM	3	26	No	T2	Positive	N1a	Positive	Negative	Positive (SYN, CHR)	X	Yes	Yes	No	175	No disease	No	No
5	51	Screen detected	X	IBC with NE features	TM	3	24	No	T2	Negative	N0	Positive	Negative	Positive (SYN, CHR)	X	Yes	Yes	No	133	No disease	No	No
6	69	Screen detected	X	IBC with NE features	TM	3	35	No	T2	Positive	N3a	Positive	Negative	Positive (SYN)	X	Yes	Yes	No	57	DOD	No	Yes (lung)
7	63	Palpable mass	X	IBC with NE features	BCS	3	38	No	T2	Positive	N1mi	Positive	Negative	Positive (SYN, CHR)	X	Yes	Yes	Yes	52	DOD	No	Yes (liver)
8	74	Palpable mass	X	IBC with NE features	BCS	3	X	X	X	X	X	Positive	Negative	X	Positive	X	X	X	X	X	X	X
9	37	Palpable mass	Negative	IBC with NE features and solid papillary growth	TM	3	132	No	T3	Positive	N1a	Positive	Negative	Positive (SYN)	Positive	Yes	Yes	No	126	AWD	No	Yes (lung)
10	62	Screen detected	X	IBC with NE features	BCS	3	20	No	T2	Negative	N0	Positive	Negative	Positive (SYN)	X	Yes	Yes	Yes	79	No disease	No	No
11	59	Screen detected	X	IBC with NE features	TM	3	15	No	T1c	Negative	N0	Positive	Negative	Positive (SYN, CHR)	X	Yes	Yes	No	90	No disease	No	No
12	29	Palpable mass	Negative	IBC with NE features	TM	3	15	Yes	T1c	Positive	N1a	Positive	Negative	Positive (SYN, CHR)	Positive	Yes	Yes	No	86	AWD	No	Yes (liver)
13	77	Palpable mass	X	IBC with NE features	BCS	3	45	No	T2	Negative	N0	Positive	Negative	Positive (SYN, CHR)	Positive	Yes	Yes	Yes	51	DOD	No	Yes (lung)
14	47	Palpable mass	X	IBC with NE features	TM	3	15	No	T1c	Negative	N0	Positive	Negative	Positive (SYN, CHR)	X	Yes	No	Yes	83	No disease	No	No
15	63	Screen detected	X	IBC with NE features	TM	3	23	No	T2	Negative	N0	Positive	Negative	Positive (SYN, CHR)	X	Yes	Yes	No	162	AWD	No	Yes (axillary lymph node)
16	54	Screen detected	X	IBC with NE features and solid papillary growth	BCS	3	8	No	T1b	Positive	N1mi	Positive	Negative	Positive (SYN)	X	Yes	Yes	Yes	85	No disease	No	No
17	38	Palpable mass	*BRCA2*	IBC with NE features	TM	3	21	No	T2	X	X	Positive	Negative	Positive (SYN, CHR)	Positive	Yes	Yes	No	82	AWD	No	Yes (liver)
18	41	Screen detected	Negative	IBC with NE features and solid papillary growth	TM	3	35	Yes	T2	Positive	N3a	Positive	Negative	Positive (SYN)	Positive	Yes	Yes	Yes	75	AWD	No	Yes (bone)
19	74	Palpable mass	Negative	IBC with NE features and solid papillary growth	BCS	3	18	No	T1c	Negative	N0	Positive	Positive	Positive (INSM1)	Positive	Yes	Yes	Yes	60	No disease	No	No
20	50	Screen detected	Negative	IBC with NE features	BCS	3	42	Yes	T2	X	NX	Positive	Positive	Positive (CHR)	Positive	Yes	Yes	No	77	AWD	No	Yes (ovary)
21	75	Screen detected	Negative	IBC with NE features and solid papillary growth	BCS	3	8	No	T1b	X	Nx	Positive	Negative	Positive (SYN)	X	Yes	No	Yes	106	No disease	No	No
22	55	Screen detected	Negative	IBC with NE features	TM	3	16	No	T1c	Positive	N2a	Positive	Negative	Positive (SYN)	Positive	Yes	Yes	Yes	64	No disease	No	No
23	41	Palpable mass	*BRCA2*	IBC with NE features	TM	3	13	Yes	T1c	Positive	N1a	Positive	Negative	X	Positive	Yes	Yes	No	49	DOD	No	Yes (liver)
24	41	Palpable mass	Negative	IBC with NE features	BCS	3	31	No	T2	Negative	N0	Positive	Negative	Positive (SYN, CHR, INSM1)	Positive	Yes	Yes	Yes	77	AWD	No	Yes (bone)
25	45	Palpable mass	Negative	IBC with NE features	TM	3	85	No	T3	Negative	N0	Positive	Negative	Positive (SYN)	Positive	Yes	Yes	No	53	AWD	No	Yes (bone)
26	43	Screen detected	Negative	IBC with NE features	TM	3	15	No	T1c	Negative	N0	Positive	Negative	Positive (INSM1)	Positive	Yes	Yes	No	36	No disease	No	No
27	82	Screen detected	X	IBC with NE features and solid papillary growth	TM	3	23	No	T2	X	Nx	Positive	Negative	Positive (SYN, CHR)	Positive	Yes	No	Yes	157	AWD	Breast (144 months)	Yes (skin)
28	83	Screen detected	X	IBC with NE features and mucinous component	TM	3	18	No	T1c	X	Nx	Positive	Negative	Positive (INSM1)	Positive	X	X	X	X	X	X	X

Abbreviations: AWD, alive with disease; BCS, breast-conserving surgery; CHR, chromogranin; CT, chemotherapy; DOD, died of disease; ER, estrogen receptor; HER2, human epidermal growth factor receptor-2; HT, hormonal therapy; IBC-NED, invasive breast carcinomas with neuroendocrine differentiation; NACT, neoadjuvant chemotherapy; NE; neuroendocrine; RT, radiotherapy; SYN, synaptophysin; TM, total mastectomy; X, test not available/performed.
